# The Role of *ARL4C* in Erlotinib Resistance: Activation of the *Jak2*/*Stat 5*/β-*Catenin* Signaling Pathway

**DOI:** 10.3389/fonc.2020.585292

**Published:** 2020-10-28

**Authors:** Jinrong Liao, Zeng Chen, Zongyang Yu, Tao Huang, Dan Hu, Ying Su, Zhiyong He, Changyan Zou, Lurong Zhang, Xiandong Lin

**Affiliations:** ^1^Laboratory of Radiation Oncology and Radiobiology, Fujian Cancer Hospital and Fujian Medical University Cancer Hospital, Fuzhou, China; ^2^Respiratory Department, The 900th Hospital of Joint Logistic Support Force, The Chinese People's Liberation Army, Fuzhou, China; ^3^Shanghai Institute of Nutrition and Health, Chinese Academy of Sciences, Shanghai, China; ^4^Department of Pathology, Fujian Provincial Cancer Hospital and Fujian Medical University Cancer Hospital, Fuzhou, China; ^5^Department of Oncology, Fujian Cancer Hospital and Fujian Medical University Cancer Hospital, Fuzhou, China; ^6^Fujian Provincial Key Laboratory of Translational Cancer Medicine, Fuzhou, China

**Keywords:** non-small cell lung cancer, *ARL4C*, TKI resistance, β*-catenin*, *Jak*, *Stat*

## Abstract

Cancer patients who initially benefit from Erlotinib, a drug targeting *EGFR* path, eventually develop resistance to the drug. The underlying mechanism is largely unknown. This study investigated the role of *ARL4C* in Erlotinib resistance development of NSCLC. qRT-PCR and Western blotting were performed to analyze the expression of mRNA and protein of *ARL4C* in two NSCLC cell lines (HCC827 and PC-9). Several assays (MTS, colony formation, transwell migration, luciferase reporter, and chromatin-immunoprecipitation) were used to explore the role of *ARL4C* in biofunctional changes of Erlotinib-resistant cells and their associations with *Jak2/Stat 5/*β*-catenin* signaling. Results demonstrated that (1) long-term use of Erlotinib resulted in downregulation of *ARL4C*; (2) overexpression of ARL4C could regain the sensitivity to Erlotinib in the drug-resistant HCC827/ER cells, while downregulation of *ARL4C* increased HCC827, and PC-9 cells' resistance to the drug; (3) Erlotinib-induced downregulation of *ARL4C* resulted in phosphorylation of *Jak2/Stat5* and upregulation of β*-catenin* and their related molecules *Axin2, CD44, Ccnd1, Lgr-5*, and *MMP7*, which promoted the malignant behaviors of Erlotinib-resistant cells; (4) chromatin immunoprecipitation and luciferase reporter assay revealed that Stat5 could bind to β*-catenin* promoter to upregulate molecules to maintain the malignant behaviors, which might count for how Erlotinib-resistant cell survived while *EGFR* path was blocked; (5) the expression of *ARL4C* was not associated with known EGFR gene mutations in both Erlotinib-resistant cells and NSCLC tissues. Our data suggest that Erlotinib resistance of NSCLCs is associated with downregulation of *ARL4C* via affecting *Jak/Stat/*β*-catenin* signaling. *ARL4C* could serve as a biomarker to predict the effectiveness of TKI targeting therapy and a potential therapeutic target for overcoming Erlotinib resistance in NSCLC.

## Introduction

Lung cancer is the most commonly diagnosed malignancy and a leading cause of cancer death worldwide (1). Non-small cell lung cancer (NSCLC) accounts for ~80% of all lung cancer cases (2). *EGFR* (Epidermal Growth Factor Receptor) tyrosine kinase inhibitors (TKIs) are a group of important targeting drugs for the treatment of NSCLCs with *EGFR* mutations, including exon 19 deletions or L858R substitutions. However, the acquired drug resistance occurs within 1 year after the treatment with the first generation of EGFR-TKI, which is related partially with the T790M secondary gatekeeper mutation (3), or activation of other alternative pathways, such as *HGF/Met* (hepatocyte growth factor/mesenchymal-epithelial transition factor), *HER2* (Erb-B2 Receptor Tyrosine Kinase 2), and *PIK3CA* (Phosphatidylinositol-4,5-Bisphosphate 3-Kinase Catalytic Subunit Alpha) (4). However, mechanisms of TKI resistance in about 30% cases remain unclear (5). Seeking for the unknown mechanisms is a hot research topic in lung cancer research (6).

With selected methods, we found that downregulation of *ARL4C* (ADP-ribosylation factor-like 4C) might be associated with TKI resistance. *ARL4C*, also known as arl7, is a 192-amino-acid protein belonging to a small GTP enzyme. It is one of the subfamily members of ADP ribosylation factor and plays an important role in vesicle transport and signal transduction (7). The biofunction of *ARL4C* is not very clear yet. A study conducted by Su and his colleagues showed that high-level expression of *ARL4C* could inhibit the migration of ovarian cancer cells. Patients with high level of *ARL4C* mRNA had a good prognosis (8). Another study with gastric cancer showed that the expression of *ARL4C* was abnormal and the molecule was involved in tumor cell growth and cell migration (9). Using immunohistochemical analysis, Fujii et al. showed that *ARL4C* was abnormally expressed in lung cancer tissues. It was involved in the proliferation and invasion of lung cancer cells *in vitro* and *in vivo* (10). However, the role of *ARL4C* in TKI resistance is unexplored.

In this study, we investigated the role of *ARL4C* in TKI resistance of NSCLC cells by analyzing its functions with various assays. Our data demonstrated for the first time that *ARL4C* contributed to TKI resistance by activation of the *JAK*/*STAT* (Janus Kinase/signal transducer and activator of transcription) signaling pathway for survival when *EGFR* path blocked. The results suggest that *ARL4C* could be a promising biomarker for patients who likely benefit from TKI-based targeting therapy.

## Methods

### NSCLC Tissue Samples

NSCLC tissues from 42 patients (22 men and 20 women with a median age of 60.5 years old) who have undergone lung cancer resection in Fujian Cancer Hospital between January 2008 and June 2009 were collected. None of the patients received chemotherapy before surgery, and cancer tissues were obtained immediately after surgical resection. One part of every cancer specimen was frozen at −80°C for measuring *ARL4C* mRNA level. The remaining part of the tissues was fixed in formalin and embedded in paraffin (FFPE) for detecting EGFR mutations.

### Selection of TKI Erlotinib-Resistant Cell Line

HCC827 and PC-9 (*EGFR* 19del) cell lines purchased from ATCC were cultured in H1640 medium, containing 10% FBS (GIBCO BRL, Rockville, MD, U.S.A. Cat No. 10099233), 100 U/ml penicillin, and 100 μg/ml streptomycin in an incubator containing 5% CO_2_ at 37°C. The cells were exposed to gradually increased concentration of Erlotinib (Selleck, Houston, TX, USA. Cat No. S1023) from 0, 100, 200, 400, 800, 1,600, and 3,200 nM in their culture medium. After passing 17 generations in 6 months of the selection, Erlotinib was removed from the medium. The cells growing in 3,200 nM of Erlotinib were labeled as TKI Erlotinib-resistant (ER) cell lines HCC827/ER and PC-9/ER, respectively.

### *ARL4C* Overexpression or Knockdown Cell Lines

Several virus vectors were purchased from Hanheng Biotechnology Co Ltd (Beijing, China). To create cell lines with *ARL4C* overexpression, *ARL4C* expression vector HBLV-ARL4C (pHBLV-CMV-mcs-3flag-EF1-ZsGReen-T2A-PURO inserted with *ARL4C* gene) was used. The original vector was used as vector alone control. For *ARL4C* knockdown in cells, HBLV-ARL4C-shrna1, HBLV-ARL4C-shrna2, and HBLV-ARL4C-shrna3 were used and their parental vector *pHBLV-U6-MCS-CMV-Zsgreen* was used as vector alone control. HCC827, HCC827/ER, PC-9, and PC-9/ER cells cultured in six-well-plates (5 × 10^5^/well) were infected with 10 MOI of respective viral vectors in the presence of 6 μg/ml of polyamine. After 48 h, the cells were selected with 2 μg/ml of Puromycin for 2 weeks to obtain stable infected cells. Newly established cells were HCC827/ER/vector, HCC827/ER/ARL4C-OE, HCC827/vector, HCC827/ARL4C-SH, PC-9/ER/vector, PC-9/ER/ARL4C-OE, PC-9/vector, and PC-9/ARL4C-SH. The symbols -OE and -SH means overexpression and knockdown, respectively.

### Detection of mRNA Levels of *ARL4C, β-Catenin, Axin2, CD44, Ccnd1, Lgr-5*, and *MMP-7*

The level of ARL4C mRNA expression was determined in 42 lung cancer tissues by real-time PCR. ARL4C and β-atenin, *Axin2* (Axis Inhibition Protein 2), *CD44, Ccnd1* (Cyclin D1), *Lgr-5* (Leucine Rich Repeat Containing G Protein-Coupled Receptor 5), and *MMP-7* (Matrix Metallopeptidase 7) were assessed in NSCLC cell lines (HCC827, PC-9, HCC827/ER, and PC-9/ER) also by real-time PCR. Total RNA was extracted from tissues and cell lines using Trizol reagent (Invitrogen, Grand Island, NY, USA) following the manufacturer's instruction. cDNA was synthesized from 1 μg of total RNA using M-MuLV reverse transcriptase (Promega, Madison, WI, USA). Real-time PCR was performed using SYBR1 Green Dye detection systems (Roche, Switzerland). The primers for amplifying *ARL4C*, β*-catenin, Axin2, CD44, Ccnd1, Lgr-5*, and *MMP-7* are shown in Supplementary Table 1. Real-time PCR parameters were 95°C for 10 min, followed by 40 cycles of 95°C for 10 s, 55°C for 10 s and 72°C for 20 s, and 40°C for 30 s at the end of the 40 cycles. Relative quantity of mRNA expression was calculated by using the 2^−ΔΔCt^ method. All measurements were repeated in triplicate.

Relative mRNA expression levels of *ARL4C* were quantified in all lung cancer samples using the comparative 2^−ΔΔCt^ method and lung cancer sample T27 as reference reported previously (11, 12). The housekeeping gene glyceraldehyde phosphate dehydrogenase (*GAPDH*) was used to normalize expression levels of *ARL4C*.

### Protein Levels of ARL4C, β-Catenin, and JAK/STAT Signaling

Cells were harvested and lysed using RIPA buffer (50 mM Tris–Cl, pH 8.0, 150 mM NaCl, 5 mM EDTA, 0.1% SDS, and 1% NP-40) supplemented with protease inhibitor cocktail (Abcam, Cambridge, MA, USA. Cat No. ab65621). The cell lysates were centrifuged at 12,000 rpm for 30 min at 4°C. Supernatants were collected and protein concentrations were determined by BCA protein assay (Thermo Scientific, Rockford, Illinois, USA). The supernatants (each with 25 μg proteins) were electrophoresed on 10–12% polyacrylamide gel with sodium dodecyl sulfate (SDS) and then transferred onto nitrocellulose membranes (Millipore, Burlington, MA, USA) at 100 V for 1.5 h. After blocking with 3% BSA in TBST (TBS−1% Tween 20) for 1 h, the membranes were incubated with primary antibodies of ARL4C (1:500. Abcam, Cambridge, UK, Cat No. ab122025), and 1:1,000 diluted antibodies against β-catenin (CST, Danvers, MA, USA. Cat No. 8480), p-JAK 2 (CST, Danvers, MA, USA. Cat No. 4406), and p-STAT5 (Cell Signaling Technology, Danvers, MA, USA. Cat No. 4322) overnight at 4°C, respectively. After wash, the membranes were further incubated with horseradish peroxidase-conjugated anti-rabbit antibody. Finally, protein bands were developed with the enhanced chemiluminescence Western blot detection kit Immobilon ECL Ultra Western HRP Substrate (Millipore, Bedford, MA, USA. Cat No. WBULS0500), and images were captured on image station 4,000 mm pro (Carestream, Canada). Image J program was used to quantify the protein band intensity relative to loading control of β-actin.

### Effects of Erlotinib on Cell Proliferation and IC50

Cells were plated into 96-well-plates (5,000 cells/well) and grew overnight. Different concentrations of Erlotinib (0, 100, 200, 400, 800, 1,600, and 3,200 nM) were added into the cell cultures in triplicate. After culturing for 72 h, 20 μl of MTS (Promega, Madison, WI, USA. Cat No. g3582) and 100 μl of serum containing medium were added to each well-followed by incubation at 37°C for 2 h. H1640 only with serum was used as background control. The absorption of the palates at 490 nm was read on a Bio-Rad (Hercules, CA, USA) plate reader (model 680). The proliferation of Erlotinib-treated cells was normalized with the control cells (no Erlotinib). IC50 was calculated with SPSS17.0. The experiment was repeated three times.

### Colony Formation Assay

For colony-formation assay, cells (about 500 cells/well) were seeded and grew in six-well-plates for 48 h before adding Erlotinib. After 14 days, the cells were fixed in methanol and stained with 0.2% crystal violet. Cell colonies (>50 cells/colony) were pictured with Image Scanner (GE, Piscataway, NJ, USA). The number of colonies in each well was counted using *Image J*.

### Transwell Invasion Experiment

Cell concentration was adjusted to 7 × 10^5^/ml, and 100 μl of the cell suspension was placed onto the upper chamber of each well on 24-well-Transwell plates coated with 1 mg/ml fibronectin (Millipore). The lower chamber contained medium with 20% FBS. After incubation at 37°C for 48 h, the cells in the upper chamber were wiped off with cotton swabs, and the cells in the other side of chamber membrane were fixed with methanol for 15 min, dried, stained with 0.1% crystal violet, and randomly pictured with a magnification of ×200 under inverted microscope (Olympus, Japan). The cells were counted with *Image J* program and the average number of the cells from 15 fields was used. The experiment was repeated three times.

### Luciferase Assays

Using the online transcription factor binding sites (TFBS) software (http://alggen.lsi.upc.es), we predicted the *CTNNB1* promoter containing a *STAT5A* binding site. *CTNNB1* (β*-catenin*) promoter (−200 to 0 regions) was inserted into *pGL3-basic vector* (Promega, Madison, WI, USA) as *pGL3-CTNNB1* luciferase reporter plasmid (wild-pGL3-CTNNB1). The STAT5 site of the *CTNNB1* promoter was mutated (from 5′-atttttctgtcag-3′ to 5′-taaaaagacagtc-3′) and was cloned into *pGL3-basic vector* to generate *CTNNB1* promoter mutated reporter plasmid mut-pGL3-CTNNB1. All constructs were verified by sequencing. For the luciferase reporter assay, HEK293T cells, HEK293T/ARL4C-SH, HCC827, and HCC827/ARL4C-SH cells were transfected with *pGL3-CTNNB1-Luc* or *mut-pGL3-CTNNB1* using X-treme GENE HP DNA Transfection Reagent (Roche, Basel, Switzerland. Cat No. 6366236001). Renilla luciferase was used as internal control. Forty-eight hours later, the transfected cells were harvested and the luciferase activity was measured by Dual-luciferase Reporter Assay System (Promega Corporation, Madison, WI, USA). The relative firefly luciferase activity was calculated by normalizing transfection efficiency using the Renilla luciferase activity.

### Chromatin-Immunoprecipitation (ChIP)

ChIP assay was carried out using a SimpleChIP Enzymatic Chromatin IP Kit (Cell Signaling Technology, Beverly, MA, USA, Cat No. 9002) following the manufacturer's instruction. Briefly, 5 × 10^6^ cells were fixed with 1% formaldehyde and quenched in 0.125 M glycine. Cells were sonicated by Bioruptor Sonication System UCD-300. DNA was immunoprecipitated by either control IgG or phospho-stat5 antibody. Precipitated DNA samples and inputs were amplified by PCR. The primers used for the amplification of stat5 binding site in β-catenin promoter are 5′-cctcttccccgttgtttcca-3′ (sense) and 5′-ggggtgattctttgctaatttca-3′ (antisense).

### Detection of *EGFR* Mutations in Both NSCLC Samples and Cell Lines

Two methods were used to detect *EGFR* mutations. For 42 NSCLC paraffin specimens, DNA was obtained using a paraffin tissue DNA Extraction kit (Qiagen, Hilden, Germany. Cat No. 56404). The concentration of DNA was adjusted to 1 ng/ml, and EGFR mutations were detected using the amplification refractory mutation system (ARMS) with human EGFR Mutations Detection kit (Amoy Diagnostics, Xiamen, China. Cat No. ADx-EG01) according to the manufacturer's protocol as previously described (13). Briefly, ARMS-PCR assay was performed in a 50-μl volume containing 5 μl of PCR buffer, 10 pM forward and reverse primers, 20 pM probe, and 12.5 mM dNTPs. The thermocycling conditions were as follows: 95°C for 5 min, then 15 cycles of 95°C for 25 s, 64°C for 20 s, and 72°C for 20 s, followed by 31 cycles of 93°C for 25 s, 60°C for 35 s, and 72°C for 20 s.

To determine if there is any association between *ARL4C* expression and *EGFR* mutations, eight cell lines with different levels of *ARL4C* were examined with the next-generation sequencing (NGS). DNA was extracted using the GONOROAD kit (Qiagen, Hilden, Germany) and 200 ng of DNA was used to build the library using NEBNext Ultra II DNA library Prep Kit for Illumina (NEB, Ipswich, MA, USA). Integrated DNA technologies (IDT, Skokie, IL, USA) customized probes were used for hybridization capture. All libraries were performed on an MGISEQ2000 instrument according to the manufacturer's instructions (BGI, Shenzhen, Guangdong, China) with 200 cycles, standing for paired-end 100 bp. Then, IDT 10-hotspot gene panels from all eight libraries were used, which included *ALK, BRAF, EGFR, ERBB2, KRAS, MET, NRAS, PIK3CA, RET*, and *ROS1*, and were quantitated using Library Quantification Kit-Illumina/Universal (Kapa Biosystems, Wilmington, MA, USA) on an ABI 7500 Real Time PCR system (Applied Biosystems, Waltham, MA, USA). The mutations were detected by the following methods: the Trimmomatic (version 0.39, parameter: PE -threads 4 -phred33 ILLUMINACLIP: adapter.fa:2:30:10 MINLEN:15), and the adapter sequences were AAGTCGGAGGCCAAGCGGTCTTAGGAAGACAA and AAGTCGGATCGTAGCCATGTCGTTCTGTGAGCCAAGGAGTTG and were used to narrow down the raw sequencing data (Fastq), filtering out the adapter contamination reads and low-quality reads to get clean data. Bwa Aln (Version: 0.7.12-r1039) algorithm was used to align the clean data of the human reference genome (hg19) and to get the Sequence Alignment/Map format (sam) file. For the Binary Alignment/Map format (bam) file, the sam file was sorted and the artificial duplication reads were removed by samtools (Version: 0.1.19-44428cd). According to the bed interval file of the 10-hotspot gene panels, Freebayes (version: v1.0.2-6-g3ce827d, parameter: -j -m 10 -q 30 -F 0.001 -C 1 -t bed.file –f hg19.fa) was used to determine the single-nucleotide polymorphisms (SNPs) and insertions or deletions (indels) and then ANNOVAR was used for the annotation.

### Statistical Analysis

All data were presented as mean ± SD. Student's *t*-test was used for analysis. ANOVA was used to determine the statistical difference between or among different experimental groups. The value *P* < 0.05 was considered as statistically significant difference.

## Results

### TKI Resistant NSCLC Cell Lines Expressed a Low Level of *ARL4C*

**Figure 1:**
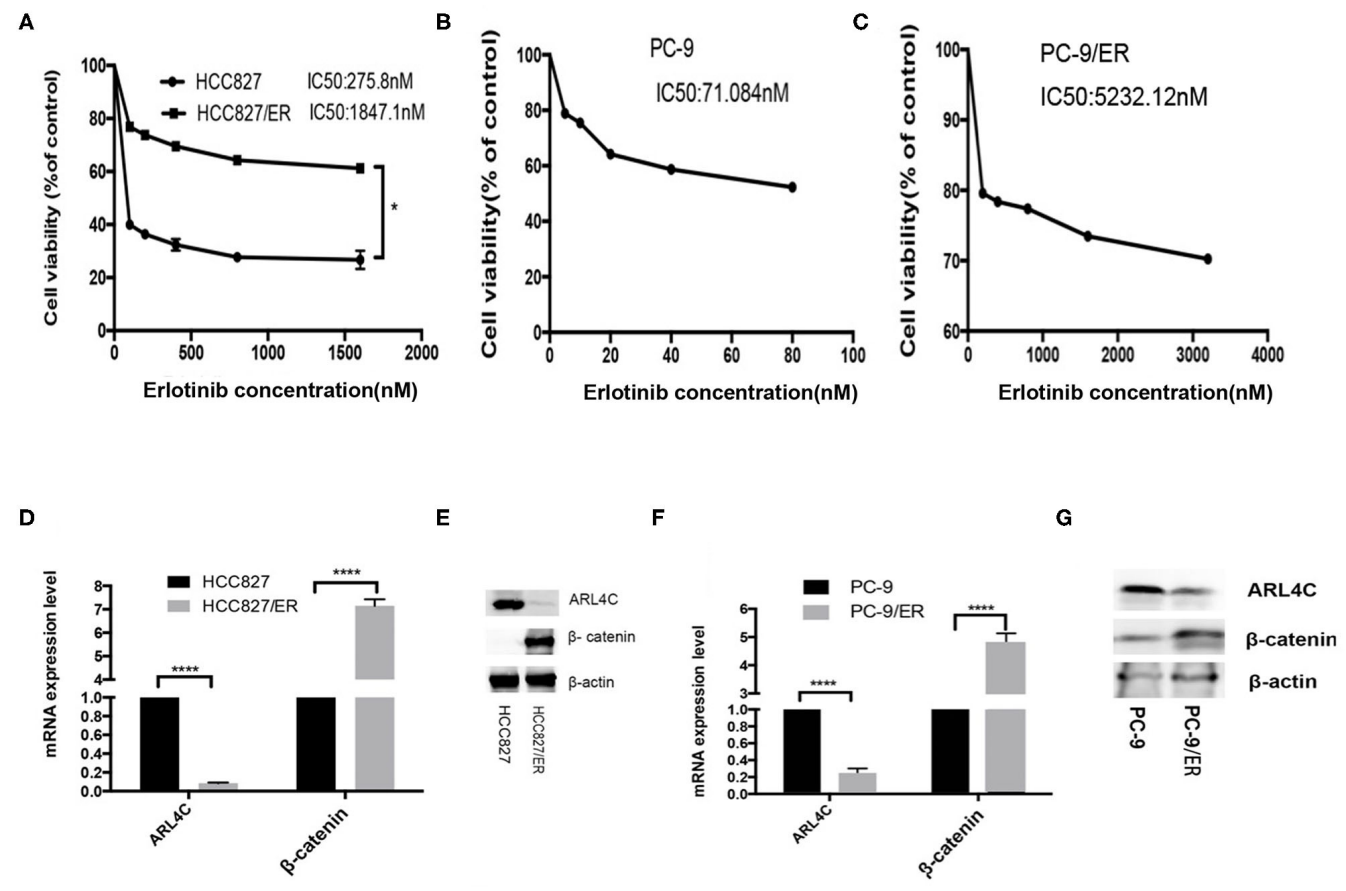
Expression of *ARL4C* and β*-Catenin* in TKI-resistant NSCLC cell lines. **(A)** MTS assay of the proliferation of in Erlotinib-resistant HCC827/ER and Erlotinib-sensitive HCC827 cells in different concentrations of Erlotinib. **(B,C)** MTS assay of the dose response of Erlotinib-sensitive PC-9 cells **(B)** and Erlotinib-resistant PC-9/ER **(C)** to different concentrations of Erlotinib. **(D,E)** qPCR and Western blotting analyzed *ARL4C* and β-catenin expression in HCC827/ER and HCC827 cells. **(F,G)** qPCR and Western blotting detected *ARL4C* and β*-catenin* expression in PC-9/ER and PC-9 cells. *P < 0.05; ****P < 0.0001.

Two NSCLC cell lines, HCC827 and PC-9, were subjected to Erlotinib selection in culture. After 6 months, IC50 of Erlotinib was significantly increased from 289 to 1,843 nM for HCC827 and from 71.08 to 5232.12 nM for PC-9. These Erlotinib-resistant (ER) cell lines were named HCC827/ER and PC-9/ER, which were 5.37 and 73.60 times more resistant to Erlotinib than their parental cells, respectively (Figures 1A–C). However, results of qPCR and Western blotting demonstrated that the levels of *ARL4C* were significantly lower in HCC827/ER and PC-9/ER cells than in their parental cells (Figures 1D–G, *P* < 0.0001). Furthermore, β*-Catenin* expression significantly increased in HCC827/ER and PC-9/ER, compared with their parental cells (Figures 1D–G, both *P* < 0.0001). Collectively, these results showed that the expression of *ARL4C* was reduced in TKI-resistant cells (HCC827/ER, PC-9/ER), while the expression of β*-catenin* was elevated in TKI-resistant cells.

### Knocking Down *ARL4C* Resulted in Enhancing Erlotinib Resistance and Increasing Cell Migration

**Figure 2:**
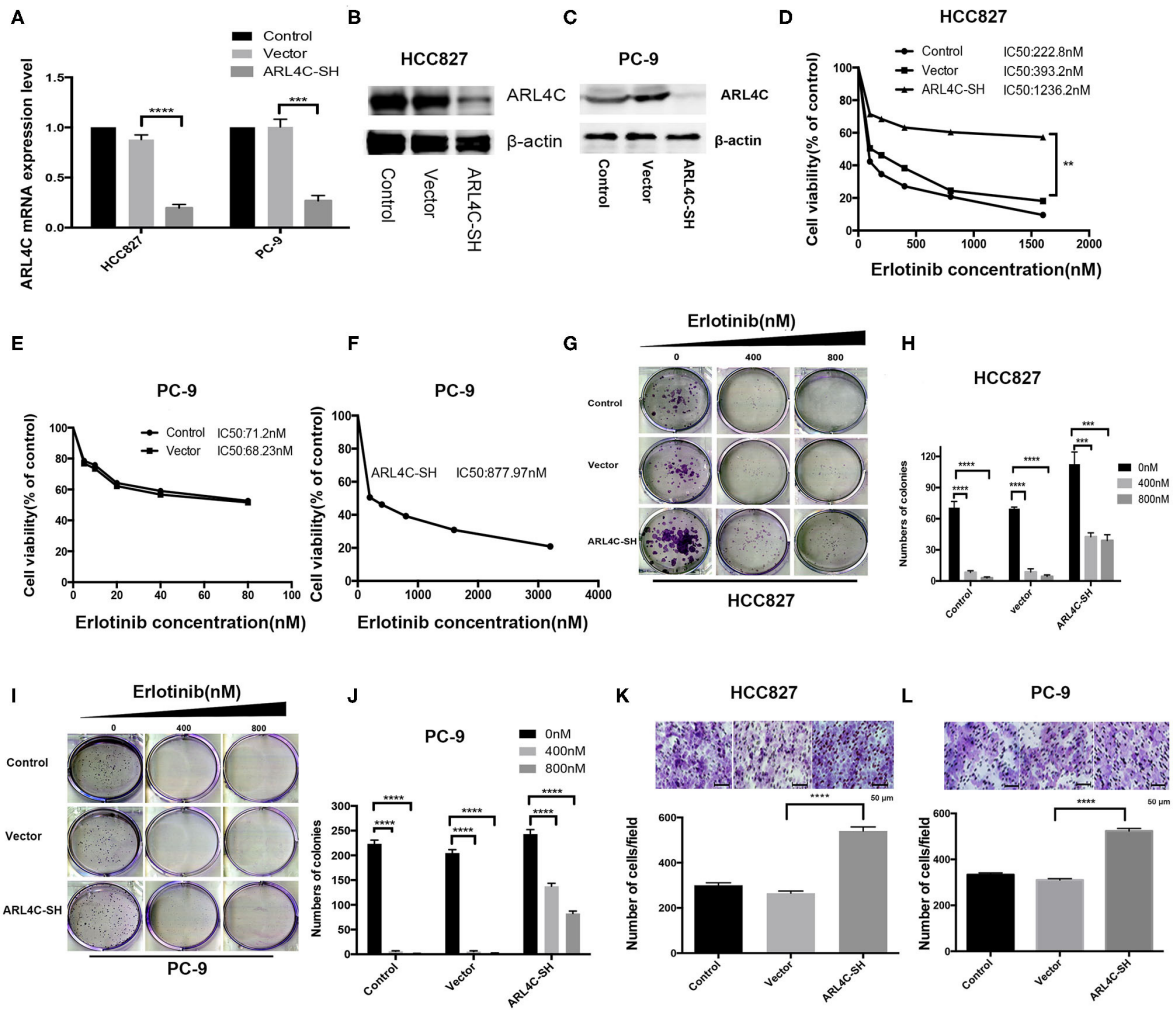
*ARL4C* knockdown enhances Erlotinib resistance of lung cancer cells. **(A)** qPCR quantification of *ARL4C* mRNA levels in *ARL4C* knockdown cells HCC827 (HCC827-ARL4C-SH) and PC-9 (PC-9-ARL4C-SH), control vector-infected HCC827 (HCC827-Vector) and PC-9 (PC-9-Vector), and their parental HCC827 and PC-9 control cells. **(B)** Western blotting detection of *ARL4C* expression in HCC827-ARL4C-SH, HCC827-Vector, and HCC827 (control) cells, and in **(C)** in PC-9-ARL4C-SH, PC-9-Vector, and PC-9 (control) cells. **(D)** IC50 of HCC827-ARL4C-SH, HCC827-Vector, and HCC827 (control) cells measured by MTS. **(E,F)** Erlotinib IC50 of PC-9-ARL4C-SH, PC-9-Vector, and PC-9 (control) cells measured by MTS. **(G,H)** Colony formation of HCC827-ARL4C-SH, HCC827-Vector, and HCC827 (control) cells. **(I,J)** Colony formation of PC-9-ARL4C-SH, PC-9-Vector, and PC-9 (control) cells. **(K)** Transwell assays of the migration of HCC827-ARL4C-SH, HCC827-Vector, and HCC827 (control) cells. **(L)** Transwell assays of the migration of PC-9-ARL4C-SH, PC-9-Vector, and PC-9 (control) cells. **P < 0.01; ***P < 0.001; ****P < 0.0001.

To confirm that the downregulation of *ARL4C* was associated with the increased Erlotinib resistance, sh-ARL4C-knockout lentivirus was used to infect HCC827 and PC-9 cells. Western blotting showed that the expression level of *ARL4C* in the infected cells HCC827/ARL4C-sh and PC-9/ARL4C-sh were significantly lower than those in control cells infected with lentivirus vector alone (HCC827/Vector and PC-9/Vector) (Figures 2A–C, both *P* < 0.0001).

Effect of low *ARL4C* on Erlotinib IC50 was examined with MTS assay. Results showed that knockdown of *ARL4C* significantly increased Erlotinib resistance as compared to their parental cells. IC50 values of Erlotinib for HCC827, HCC827/vector, and HCC827/ARL4C-sh were 222.8, 393.2, and 1236.2 nM, respectively. The values for PC-9, PC-9/vector, and PC-9/ARL4C-sh were 71.2, 68.23, and 877.97 nM, respectively (Figures 2D–F, *P* < 0.01).

The cells with knockdown of *ARL4C* (HCC827/ARL4C-sh and PC-9/ARL4C-sh) had a marked enhancement of proliferation and colony formation, compared to their parental HCC827 and PC-9 cells when treated with Erlotinib (Figures 2G–J, both *P* < 0.001). These *ARL4C* low-expressing cell lines HCC827/ARL4C-sh and PC-9/ARL4C-sh cells also had a significantly increased capability of the migration, passing through the polyester membrane, compared with control cells HCC827/vector and PC-9/vector (Figures 2K,L, both *P* < 0.001).

These results suggest that knockdown *ARL4C* enhances TKI Erlotinib tolerance, colony formation, and migration of NSCLC cells.

### Overexpression of *ARL4C* in Erlotinib-Resistant HCC827/ER and PC-9/ER Cells Inhibited Their Tolerance to Erlotinib and Cell Migration

**Figure 3:**
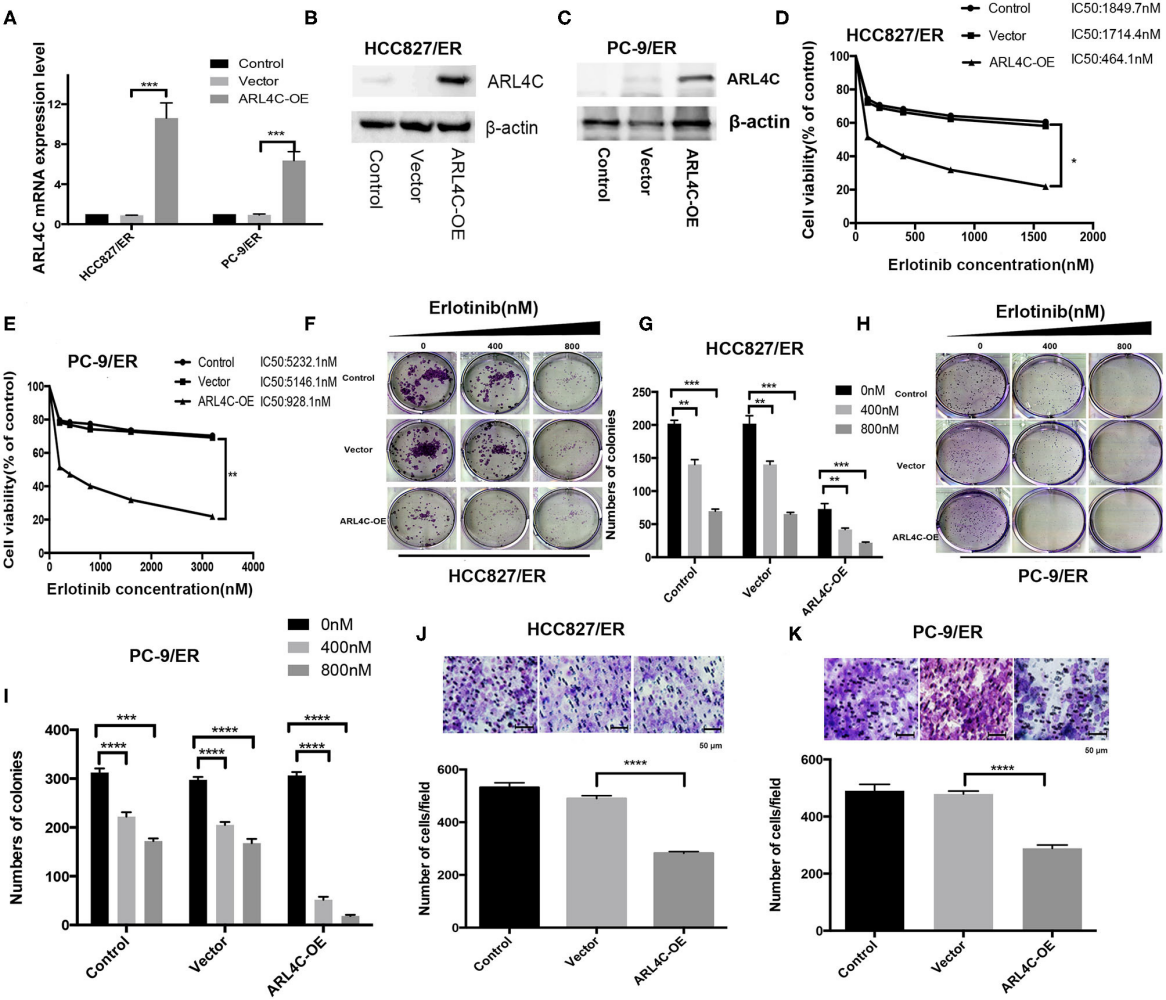
*ARL4C* overexpression decreases Erlotinib resistance of lung cancer cells. **(A)** Left: qPCR quantification of *ARL4C* expression in HCC827/ER ARL4C-overexpressing (HCC827/ER-ARL4C-OE), HCC827/ER-Vector (HCC827/ER-Vector), and HCC827/ER (control) cells. Right: qPCR quantification of *ARL4C* expression in PC-9/ER ARL4C-overexpressing (PC-9/ER-ARL4C-SH), PC-9/ER-ARL4C-control (PC-9/ER-Vector), and PC-9/ER (control) cells. **(B)** Western blotting detection of *ARL4C* expression in HCC827/ER-ARL4C-OE, HCC827/ER-Vector, and HCC827/ER (control) cells. **(C)** Western blotting detection of *ARL4C* expression in PC-9/ER-ARL4C-OE, PC-9/ER vector, and PC-9/ER (control) cells. **(D,E)** Erlotinib IC50 for HCC827/ER and PC-9 /ER cells ARL4C overexpressing with HBLV-ARL4C-SH3 and Vector measured by MTS. **(F,G)** Colony formation of HCC827/ER-ARL4C-OE, HCC827/ER-Vector, and HCC827/ER (control) cell. **(H,I)** Colony formation of PC-9/ER-ARL4C-OE, PC-9/ER-vector, and PC-9/ER (control) cells. **(J)** Transwell assay of the migration of HCC827/ER-ARL4C-OE, HCC827/ER-Vector, and HCC827/ER (control) cells. **(K)** Transwell assay of the migration of PC-9/ER-ARL4C-OE, PC-9/ER-vector, and PC-9/ER (control) cells. *P < 0.05; **P < 0.01; ***P < 0.001; ****P < 0.0001.

To further confirm the relationship between Erlotinib resistance and *ARL4C* expression, both Erlotinib-resistant HCC827/ER and PC-9/ER cells were infected with *ARL4C* overexpression lentivirus to regain the *ARL4C* expression. Results of qPCR and Western blot showed that the expression levels of *ARL4C* in HCC827/ER/ARL4C-OE and PC-9/ER/ARL4C-OE were significantly higher than those of vector alone control cells, HCC827/ER/Vector, and PC-9/ER/Vector (Figures 3A–C, *P* < 0.001).

To examine the effect of high level of *ARL4C* on cell susceptibility to Erlotinib, the IC50 was measured with MTS assay. Results showed that the overexpression of *ARL4C* significantly decreased Erlotinib resistance of both HCC827/ER and PC-9/ER cells. IC50 values of Erlotinib for HCC827/ER, HCC827/ER/Vector, and HCC827/ER/ARL4C-OE cells were 1.85, 1.71, and 0.47 μM, respectively. The values for PC-9/ER, PC-9/ER/Vector, and PC-9/ER/ARL4C-OE cells were 5232.1, 5146.1, and 928.1 nM, respectively (Figures 3D,E, both *P* < 0.05).

Similarly, the effect of high level of *ARL4C* on cell colony formation and migration in the presence of Erlotinib was tested. Results showed that the overexpression of *ARL4C* in HCC827/ER/ARL4C-OE and PC-9/ER/ARL4C-OE cells resulted in an reduced sensitivity to Erlotinib as compared to HCC827/ER and PC-9/ER cells (Figures 3F–I, *P* < 0.01, *P* < 0.001, and *P* < 0.0001). The migration of HCC827/ER/ARL4C-OE and PC-9/ER/ARL4C-OE was significantly decreased. The number of cells passing through the polyester membrane decreased, compared to control cells HCC827/ER-vector and PC-9/ER-vector (Figures 3J,K, both *P* < 0.0001).

These data suggest that overexpression of *ARL4C* in Erlotinib-resistant lung cancer cells increases the sensitivity of the cells to TKI Erlotinib and inhibits cell migration.

### Enhance of *β-Catenin* Is Critical for ARL4C-Associated Erlotinib Resistance

It has been shown that cells resistant to Erlotinib expressed a high level of β-catenin (12). Our results also showed that *ARL4C* was expressed at a significantly low level in Erlotinib-resistant HCC827/ER and PC-9/ER cells, compared to Erlotinib-sensitive HCC827 and PC-9 cells (Figures 1D–G, *P* < 0.0001), while β*-Catenin* expression significantly increased in Erlotinib-resistant cells (Figures 1D–G, *P* < 0.0001). Therefore, we speculate that β*-catenin* might contribute to *ARL4C*-associated Erlotinib resistance.

**Figure 4:**
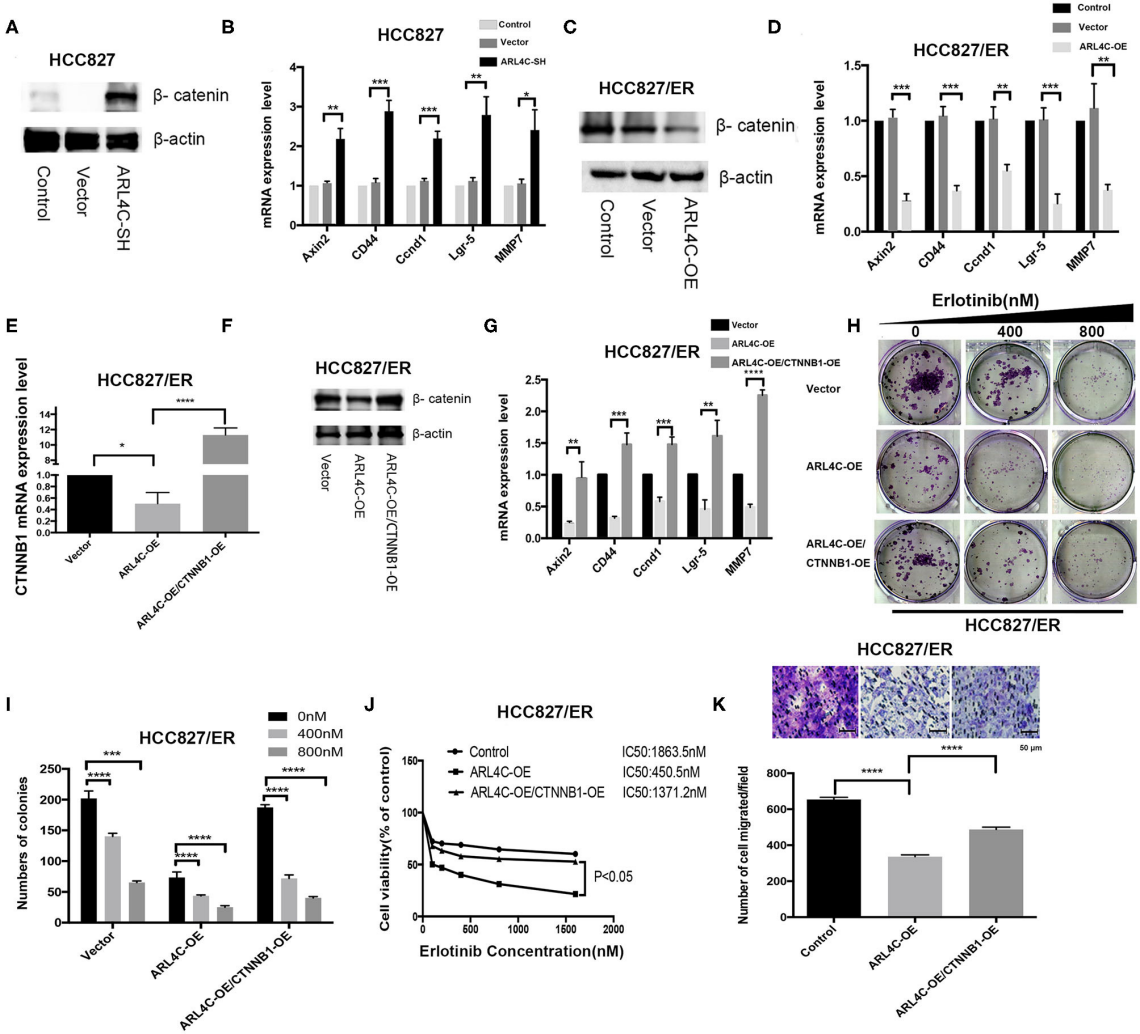
Enhancement of β*-catenin* is critical for *ARL4C* to reduce TKI Erlotinib resistance. **(A)** Western blot showed β-catenin protein expression in HCC827-ARL4C-SH, HCC827-Vector, and HCC827 (control) cells. **(B)** The expression of β*-catenin*-target genes *Axin, CD44, Ccnd1, Lgr-5*, and *MMP7* in HCC827-ARL4C-SH, HCC827-Vector, and HCC827 (control) cells detected by real-time PCR. **(C)** Detection of β-catenin protein expression in HCC827/ER-ARL4C-OE, HCC827/ER-Vector, and HCC827/ER (control) cells by Western blot. **(D)** The expression of β*-catenin*-target genes *Axin2, CD44, Ccnd1, Lgr-5*, and *MMP7* in HCC827/ER-ARL4C-OE, HCC827/ER-Vector, and HCC827/ER (control) cells measured by real-time PCR. **(E,F)** β*-catenin* (*CTNNB1*) expression in HCC827/ER/ARL4C-OE, HCC827/ER/ARL4C-OE–vector, and HCC827/ER/ARL4C-OE-CTNNB1-OE in the rescue assay analyzed by real-time PCR and Western blot. **(G)** Real-time PCR quantified the expression of β*-catenin*-target genes, *Axin2, CD44, Ccnd1, Lgr-5*, and *MMP7* in HCC827/ER/ARL4C-OE, HCC827/ER/ARL4C-OE–vector, and HCC827/ER/ARL4C-OE-CTNNB1-OE. **(H,I)** Colony formation of HCC827/ER/ARL4C-OE, HCC827/ER/ARL4C-OE–vector, and HCC827/ER/ARL4C-OE-CTNNB1-OE. **(J)** Erlotinib IC50 for HCC82/ER/ARL4C-OE, HCC827/ER/ARL4C-OE–vector, and HCC827/ER/ARL4C-OE-CTNNB1-OE measured by MTS assay. **(K)** Transwell assays on the migration of HCC827/ER/ARL4C-OE, HCC827/ER/ARL4C-OE–vector, and HCC827/ER/ARL4C-OE-CTNNB1-OE. *P < 0.05; **P < 0.01; ***P < 0.001; ****P < 0.0001.

To test our hypothesis, the effect of *ARL4C* on the β*-catenin* expression in HCC827 and HCC827/ER cells was examined. As expected, knockdown of *ARL4C* with lentivirus vectors HBLV-ARL4C-shrna1s significantly increased the expression of β*-catenin* in HCC827 cells. Furthermore, the expression of β*-catenin*-regulated target genes, *Axin2, CD44, Ccnd1, Lgr-5*, and *MMP7*, was also significantly increased in *ARL4C* shRNA transfected cells (Figures 4A,B, *P* < 0.05). In contrast, overexpression of *ARL4C* significantly suppressed the expression of β-catenin as well as β-catenin-regulated target genes, *Axin2, CD44, Ccnd1, Lgr-5*, and *MMP7*, in HCC827/ER cells (Figures 4C,D, *P* < 0.01; *P* < 0.001).

To further confirm the relationship between *ARL4C* and β*-catenin*, a rescue assay was performed by overexpressing β*-catenin* in HCC827/ER/ARL4C-OE. qPCR and Western blotting showed that the expression level of β-catenin in HCC827/ER/ARL4C-OE/CTNNB1-OE was significantly higher than that in HCC827/ER/ARL4C-OE (Figures 4E,F, *P* < 0.05 and *P* < 0.0001). Moreover, the expression of ARL4C-inhibited β-catenin-target genes *Axin2, CD44, Ccnd1, Lgr-5*, and *MMP7* was increased (Figure 4G, *P* < 0.01, *P* < 0.001, and *P* < 0.0001), which was positively correlated to the expression of β*-catenin*.

In clonogenic assays, it was found that the overexpression of β*-catenin* significantly increased the proliferation of ARL4C-overexpressed HCC827/ER cells when treated with Erlotinib (Figures 4H,I, *P* < 0.001 and *P* < 0.0001). More importantly, the overexpression of β*-catenin* by lentivirus significantly decreased ARL4C-induced Erlotinib sensitivity in HCC827/ER cells. IC50 values of Erlotinib for HCC827/Vector, HCC827/ER/ARL4C-OE, and HCC827/ER/ARL4C-OE/CTNNB1-OE were 1863.5, 450.5, and 1371.2 nM, respectively (Figure 4J, *P* < 0.05). Transwell migration assay also showed that the overexpression of β*-catenin* increased the migration of *ARL4C*-overexpressed HCC827/ER cells (Figure 4K, *P* < 0.0001). These results suggest that β*-catenin* has an opposite effect to *ARL4C* on the development of TKI Erlotinib resistance of NSCLC cells.

### *ARL4C* Regulates *β-Catenin* Expression Through *JAK2/STAT5A* Signal Activation

**Figure 5:**
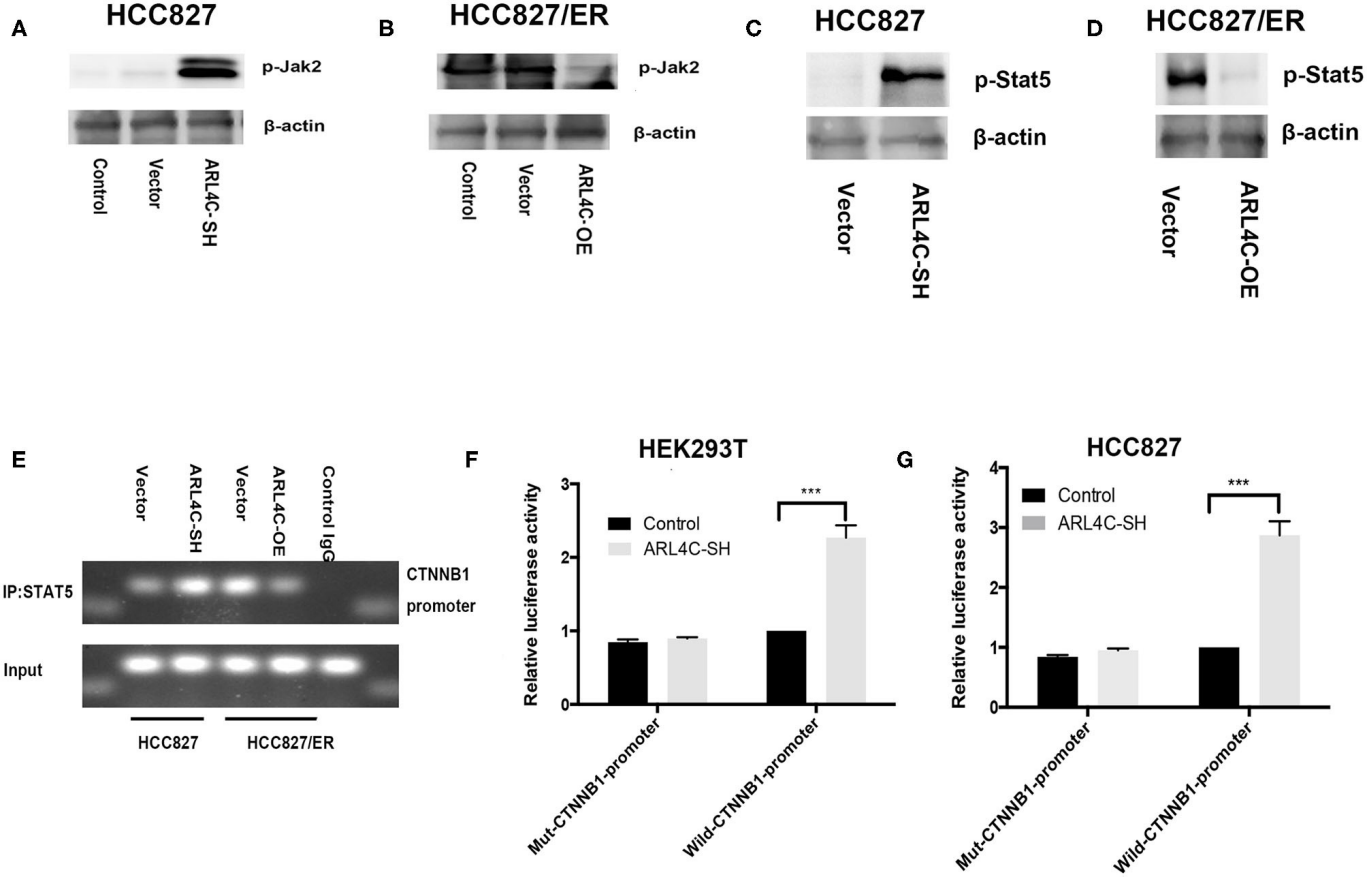
*ARL4C* regulates β-catenin expression through the *JAK2/STAT5A* signal pathway. **(A**–**D)** The expression of p-JAK2 in HCC827-ARL4C-SH, HCC827-Vector, and HCC827 (control) cells **(A)** and in HCC827/ER-ARL4C-OE, HCC827/ER-Vector, and HCC827/ER (control) cells **(B)** detected by Western blot. *STAT5* protein expression in HCC827-ARL4C-SH, HCC827-Vector cells **(C)** and in HCC827/ER-ARL4C-OE, HCC827/ER-Vector cells **(D)** also detected by Western blot. **(E)** The enrichment of the β*-catenin* (*CTNNB1*) promoter in immunoprecipitated (IP) *STAT5* and the input of *STAT5* in HCC827/ER-ARL4C-OE, HCC827/ER-Vector, HCC827-ARL4C-SH, and HCC827-Vector and followed by Western blot. Unrelavant IgG was used as a negative control staining. **(F,G)** Luciferase activity in HEK293T cells **(F)** and HCC827 cells **(G)** co-transfected with pGL3-CTNNB1-Luc or mut-pGL3-CTNNB1-Luc and HBLV-ARL4C-SH3 by the Dual-Luciferase Reporter Assay System, ***P < 0.001.

Previous studies showed that several relevant downstream signals, such as *ERK, JAK*, and *AKT*, might be involved in tumor-promoting effects of *ARL4C* (14). To further explore potential mechanisms related to the acquired TKI resistance, phosphorylation levels of *JAK* were examined in *ARL4C* knockdown-HCC827 cells. Results showed that the phosphorylation level of *JAK2* was significantly increased, compared to that in control cells (Figure 5A). Consistently, the overexpression of *ARL4C* significantly decreased the phosphorylation of *JAK2* in HCC827/ER cells, compared to that in control cells (Figure 5B). The *JAK-STAT* signaling pathway not only regulates tumor development but also is closely related to TKI resistance (14). These results indicated that *ARL4C* abnormal expression affected the phosphorylation of *JAK2*.

We further studied if *ARL4C* regulated *STAT5A*. In HCC827 cells, the knockdown of *ARL4C* upregulated the phosphorylation of *STAT5A* as detected by Western blot analysis (Figure 5C). In contrast, the overexpression of *ARL4C* in HCC827/ER cells downregulated the phosphorylation of *STAT5A* (Figure 5D).

Chromatin immunoprecipitation showed that the overexpression of *ARL4C* resulted in the loss of the promoter of *STAT5* binding to *CTNNB1* (Figure 5E). We found that luciferase expression directed by a 200-bp fragment of the β*-catenin* promoter containing this *STAT5A* site was increased in *ARL4C* downregulated HEK293T cell (Figure 5F). Similarly, downregulation of *ARL4C* resulted in a significant enhancement of β*-catenin* promoter activity in HCC827 cells (Figure 5G). These results indicated that the β*-catenin* promoter contained a conserved *STAT5A* binding site. *STAT5A* acts as a transcription factor in regulating the mRNA expression of β*-catenin* by binding and activating the β-catenin promoter.

These data suggest that the regulation of β*-catenin* expression by *ARL4C* might be through the *JAK2/STAT5A* signal pathway in the regulating TKI resistance of NSCLC.

### Detection of *EGFR* Mutations

**Figure 6:**
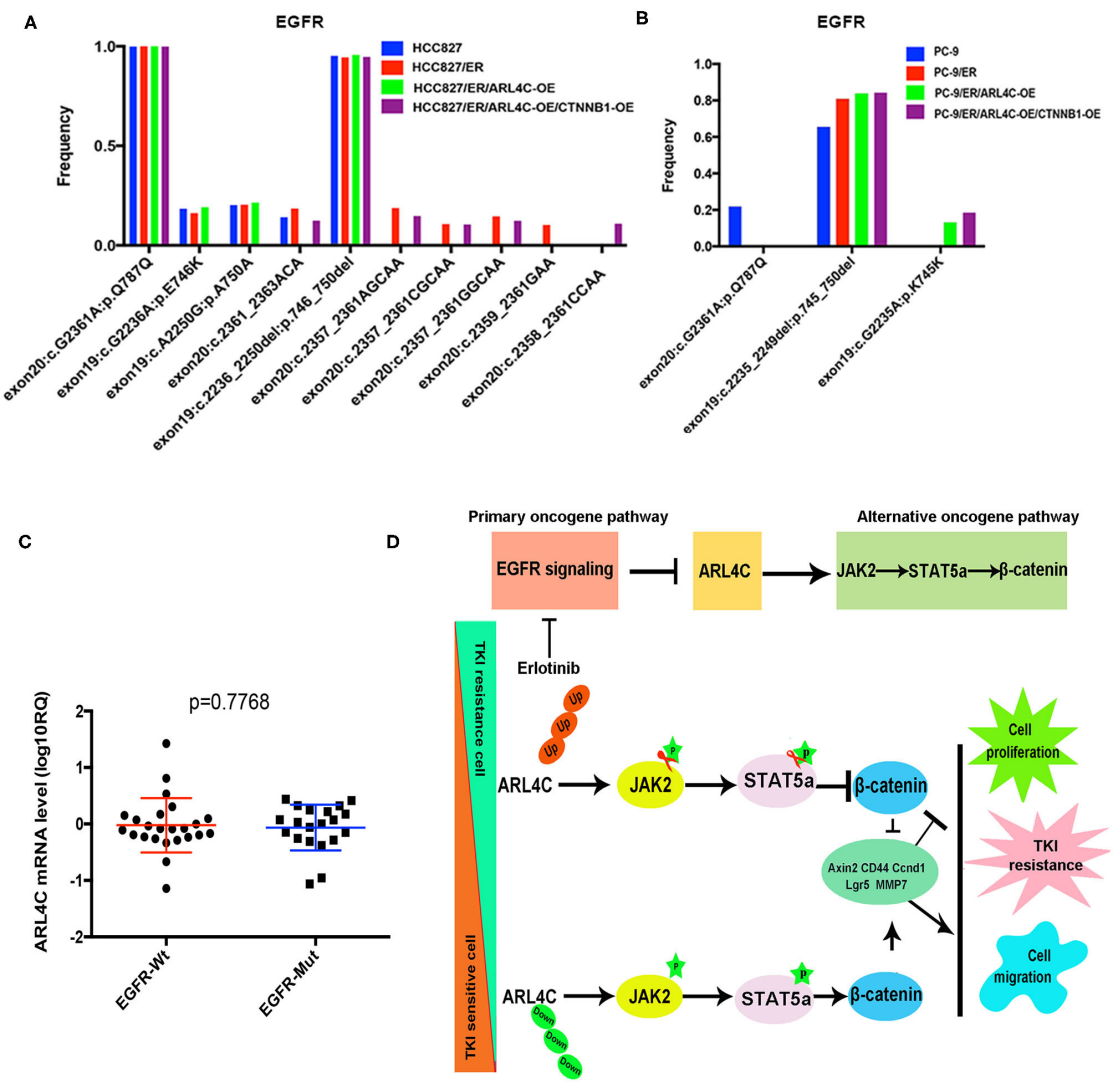
*ARL4C* alteration is not associated with known-mutated *EGFR* genes but affects *JAK/STAT/*β*-Catenin* path to stimulate the malignant behaviors of NSCLC cells: EGFR mutations were detected by ARMS with human *EGFR* Mutations Detection kit in 42 NSCLC samples. Sensitive *EGFR* mutations: Exon19:c.2236_2250del: p.746_750del; Synonymous mutation: Exon20:c.G2361A:p.Q787Q. **(A)** No differences of *EGFR* mutations among HCC827, HCC827/ER, HCC827/ER/ARL4C-OE, and HCC827/ER/ARL4C-OE /CTNNB1-OE cell lines. **(B)** No differences of EGFR mutations among PC-9, PC-9/ER, PC-9/ER/ARL4C-OE, and PC-9/ER/ARL4C-OE/CTNNB1-OE cell lines. **(C)** Distribution of ARL4C mRNA levels in NSCLC patients with EGFR mutations and without EGFR mutation(s). Bold lines represent the mean value for each patient cohort, RQ = 2^−ΔΔCt^. Similar expression level of *ARL4C* was found in 42 cases of NSCLC tissues, regardless of their *EGFR* mutation status. **(D)** A graphic illustration of the role of *ARL4C* in Erlotinib resistance. In TKI-resistant *ARL4C*-reduced NSCLC cells, the overexpression of *ARL4C* inhibited the expression of β*-catenin* by phosphorylation of *JAK/STAT*, resulting in an inhibitory effect on TKI resistance, cell proliferation, and migration, which was related to *Axin2, CD44, Ccnd1, Lgr-5*, and *MMP7*. Likewise, in the downregulation of *ARL4C* in sensitive cell lines, the results were vice versa.

To explore the association between *EGFR* mutation and *ARL4C, EGFR* mutations in 42 NSCLC paraffin samples were detected with amplification refractory mutation system (ARMS) with human *EGFR* Mutations Detection kit. Among 42, 20 were found with *EGFR* mutations and only 1 had a T790M mutation, while the other 21 had no *EGFR* mutation (in Supplementary Table 2). In these 42 tissues, mRNA expression level of ARL4C in the EGFR mutation group was −0.064 ± 0.091 (lgRQ, RQ = 2^−ΔΔCt^), while the level in the group without EGFR mutation was −0.023 ± 0.099, indicating that the expression of *ARL4C* was not associated with *EGFR* gene mutations (Figure 6C, *P* = 0.7668).

NGS was used to detect the *EGFR* mutation in eight cell lines (HCC827, HCC827/ER, HCC827/ER/ARL4C-OE, HCC827/ER/ARL4C-OE/CTNNB1-OE, PC-9, PC-9/ER, PC-9/ER/ARL4C-OE, and PC-9/ER/ARL4C-OE/CTNNB1-OE) with different levels of *ARL4C* expression. Results (Figures 6A,B, Supplementary Table 3) showed that there was a high frequency of sensitive mutation (exon19:c.2236_2250del:p.746_750del) and no T790M and C797S mutations associated with Erlotinib-induced drug-resistant cells of HCC827/ER and PC-9/ER was found. There was a high frequency of synonymous mutation (exon20:c.G2361A:p.Q787Q) in HCC827, and no new mutation was caused by the overexpression of *ARL4C* in HCC827/ER and PC-9/ER or by the overexpression of *CTNNB1* in HCC827/ER/ARL4C-OE and PC-9/ER/ARL4C-OE.

## Discussion

In this study, the underlying mechanism by which the resistance of Erlotinib, an *EGFR*-TKI, in NSCLC was explored. The study revealed for the first time that *ARL4C* was downregulated and β*-catenin* was upregulated in Erlotinib-resistant cells via *JAK2/STAT5A* signaling pathway. By manipulating the expression of these two genes (overexpression or knocking down expression), the study provided evidences that the inhibition of *ARL4C* selectively enhanced the resistance of HCC827 and PC-9 cells to Erlotinib, while the overexpression of *ARL4C* enhanced the sensitivity of the cancer cells to the drug. The regulation of the drug resistance of lung cancer cells by *ARL4C* was through activating the β*-catenin/JAK2/STAT5A* signaling pathway. These data indicate for the first time that ARL4C plays an important role in the resistance of NSCLC cells to Erlotinib.

Several mechanisms that mediate TKI resistance have been identified: (A) *EGFR* exon 20 T790M mutation (14); (B) *Met* gene amplification (15); (C) *HER2* gene amplification (16); and (D) *PIK3CA* mutation (4), which counts for the majority of gene mutations responsible for the TKI resistance. However, one third of causes of the TKI resistance remains unknown. Our data (Figure 6) showed that Erlotinib-induced *ARL4C* reduction did not associate with any currently known mutations of *EGFR*, indicating that *ARL4C* is an independent factor for the development of Erlotinib resistance.

*EGFR* path is one of the major oncogenic pathways via *Ras/Raf/MEK/ERK* signaling to promote the cancer cell proliferation, migration, and invasion. When the *EGFR* path is blocked by Erlotinib, the cancer cells have to develop another oncogenic path for their continuous survival. Our data supported that the downregulation of *ARL4C* upregulated β*-catenin* via activation of *JAK2/STAT5*, which could help the *EGFR*-path-blocked cancer cells to regain their malignant behaviors (Figure 6D).

*ARL4C* is a member of the ADP-ribosylation factor family of GTP-binding proteins. The abnormal expression of *ARL4C* possesses carcinogenic effect on many types of tumors (17). Recent studies have indicated that *ARL4C* could be induced by *EGF* and promotes the motility of cancer cells by remodeling actin cytoskeleton through *Arf6* activation (18), which might be involved in the migration, invasion, and proliferation of cancer cells (18). Consistent with this, our data showed that when *EGF* path was blocked by Erlotinib, the *ARL4C* was reduced, which triggered the upregulation of the *JAK2/STAT5/*β*-catenin* path to replace the *EGFR* oncogenic signal path. Similarly, our data supported the idea that the inhibition of *ARL4C* in wild-type HCC827 and PC-9 cells via sh-RNA could selectively enhance the Erlotinib tolerance, while the overexpression of *ARL4C* could enhance the Erlotinib sensitivity. The results indicate that *ARL4C* could be a “switch” between the *EGFR* path and the *JAK2/STAT5*/β*-catenin* path to maintain the proliferation, migration, and invasion of cancer cells when cells are exposed to Erlotinib and *EGFR* path is blocked.

It is well-known that *Wnt/*β*-catenin* signaling is a classic pathway with a crucial role in NSCLC progression. β*-catenin* is a key component in the *Wnt* signaling cascade and is involved in cancer cell proliferation and tumor progression (19). In the present study, a significant upregulation of β*-catenin* was observed in Erlotinib-resistant HCC827/ER and PC-9/ER cells compared with their parental cells HCC827 and PC-9. The result is in accordance with a previous observation that Wnt/β-catenin not only participates in the proliferation, invasion, and metastasis of tumor cells but also induces drug resistance (20). Nuclear accumulation of β*-catenin* was associated with *EGFR* mutations (21) and β*-catenin* overexpression was associated with NSCLC cell resistance to gefitinib (20). *DDX17* nucleocytoplasmic shuttling promotes acquired gefitinib resistance in NSCLC cells via activation of β*-catenin* (22). *Rab25* promotes Erlotinib resistance by activating the β*1 integrin/AKT/*β*-catenin* pathway in NSCLC (23). Alone this line, our finding added another path, *ARL4C/JAK2/STAT5/*β*-catenin*, as a new means for cancer cells to escape the survival inhibition by Erlotinib. Our data showed that in the presence of Erlotinib, the overexpression of β*-catenin* significantly increased the proliferation and decreased Erlotinib sensitivity in HCC827/ER-OE and PC-9/ER-OE (Figures 4H–K). Thus, *ARL4C*-induced Erlotinib resistance was via β*-catenin* signaling.

It is well-known that the *JAK-STAT* signaling pathway serves a crucial role in cell immunity, division, death, and tumor formation (24). The two families involved in this pathway are Janus kinases (*JAK*s) and signal transducer and activator of transcription proteins (*STATs*), encoded by the genes *JAK* (*JAK1, JAK2, JAK3*, and *TYK2*) and *STAT* (*STAT1, STAT2, STAT3, STAT4, STAT5A, STAT5B*, and *STAT6*), respectively (25). The continuous activation of *JAK/STAT* could promote malignant transformation of cells, leading to the development of cancers including NSCLC (21). The inappropriate activation of *Stat3* or *IL-7*-mediated *IL-7R-JAK3/STAT5* pathway is associated with an unfavorable prognosis in NSCLC patients and correlated with chemoresistance and radioresistance (24). Inhibition of the *JAK/STAT3* signaling pathway has therefore been recognized as a promising therapeutic strategy for NSCLC (25). In addition, it was reported that (1) the inhibition of *gp130-Jak-Stat3* signaling could partially inhibit *Wnt–*β*-catenin*–mediated intestinal tumor growth and regeneration (26); (2) *Stat3* and β*-catenin* were involved in tumorigenesis (27). By using chromatin immunoprecipitation and luciferase reporter assays, this study revealed for the first time that the promoter region of β*-catenin* contained a conserved *STAT5A* binding site. *STAT5A* acted as a transcription factor regulating the mRNA expression of β*-Catenin* by binding to and activating the β*-catenin* promoter, forming *ARL4C/JAK2/STAT5/*β*-catenin* axis for the survival of Erlotinib-resistant cells.

Our data also supported that the biofunctions of *ARL4C/JAK2/STAT5/*β*-catenin* axis were carried out by a set of molecules, such as *Axin2, CD44, Ccnd1, Lgr-5*, and *MMP7*. *Axin2* is responsible for the stability of β*-catenin* (28); *CD44* is a famous stem cell marker and adhesion molecule to enhance the proliferation, migration, and invasion (29); *Ccnd1* enhances the proliferation of cancer cells via promoting the cell's G1/S transition (30); *Lgr-5* is a biomarker for stem cells, involved in tumor development (31). *MMP7* promotes the migration and invasion of cancer cells (32). All these functional molecules were upregulated in Erlotinib-resistant cells when *ARL4C* was suppressed and *JAK2/STAT5/*β*-catenin* axis was activated (Figure 6D), serving as executors for the malignant behaviors when the *EGFR* path was blocked in NSCLC cells.

The Erlotinib stress-related *ARL4C/JAK2/STAT5/*β*-catenin* axis and the downstream *Axin2, CD44, Ccnd1, Lgr-5*, and *MMP7* could be therapeutic targets when considering how to reverse Erlotinib resistance and how to enhance the Erlotinib killing effects (Figure 6D).

## Conclusion

This study demonstrated for the first time that the *ARL4C/JAK2/STAT5/*β*-catenin* axis and their downstream molecules *Axin2, CD44, Ccnd1, Lgr-5*, and *MMP7* could help to bypass *EGFR* oncogenic path and serve as an alternative new signal/function path to maintain the survival and malignant behaviors for HCC827 and PC9 NSCLC cells under the stress of Erlotinib.

## Data Availability Statement

The datasets generated for this study can be found in online repositories. The names of the repository/repositories and accession number(s) can be found at: NCBI BioProject (PRJNA648039) (https://www.ncbi.nlm.nih.gov/Traces/study/?acc=PRJNA648039).

## Ethics Statement

The studies involving human participants were reviewed and approved by the Research Ethics Committee of the Fujian Provincial Tumor Hospital. The patients/participants provided their written informed consent to participate in this study.

## Author Contributions

XL, LZ, ZY, and YS designed the study, analyzed data, and wrote the manuscript. ZY, DH, and ZH collected and analyzed the NSCLC lung tissues for *ARL4C* expression level. JL, ZC, and CZ performed all the experiments, collected data, and created the figures. TH carried out the bioinformatics and statistical analysis. All authors contributed to the article and approved the submitted version.

## Conflict of Interest

The authors declare that the research was conducted in the absence of any commercial or financial relationships that could be construed as a potential conflict of interest.

## References

[1] Siegel RL Miller KD Jemal A Cancer statistics, 2020. CA Cancer J Clin. (2020) 70:7–30. 10.3322/caac.21590 31912902

[2] Herbst RS Morgensztern D Boshoff C. The biology and management of non-small cell lung cancer. Nature. (2018) 553:446–54. 10.1038/nature25183 29364287

[3] Wu SG Shih JY. Management of acquired resistance to EGFR TKI-targeted therapy in advanced non-small cell lung cancer. Mol Cancer. (2018) 17:38. 10.1186/s12943-018-0777-1 29455650 PMC5817870

[4] Andrews Wright NM Goss GD. Third-generation epidermal growth factor receptor tyrosine kinase inhibitors for the treatment of non-small cell lung cancer. Transl Lung Cancer Res. (2019) 8(Suppl. 3):S247–64. 10.21037/tlcr.2019.06.01 31857949 PMC6894985

[5] Majem M Remon J. Tumor heterogeneity.evolution through space and time in EGFR mutant non small cell lung cancer patients. Transl Lung Cancer Res. (2013) 2:226–37. 10.3978/j.issn.2218-6751.2013.03.09 25806236 PMC4367601

[6] Berger AH Brooks AN Wu X Shrestha Y Chouinard C Piccioni F. High-throughput phenotyping of lung cancer somatic mutations. Cancer Cell. (2016) 30:214–28. 10.1016/j.ccell.2016.06.022 27478040 PMC5003022

[7] Matsumoto S Fujii S Kikuchi A. Arl4c is a key regulator of tubulogenesis and tumourigenesis as a target gene of Wnt-beta-catenin and growth factor-Ras signalling. J Biochem. (2017) 161:27–35. 10.1093/jb/mvw069 28053143

[8] Su D Katsaros D Xu S Xu H Gao Y Biglia N. ADP-ribosylation factor-like 4C (ARL4C), a novel ovarian cancer metastasis suppressor, identified by integrated genomics. Am J Transl Res. (2015) 7:242–56. 25901194 PMC4399089

[9] Chen X Su Z Wang S Xu H. Clinical and prognostic significance of Arl4c expression in colorectal cancer. Cancer Biomark. (2016) 16:253–7. 10.3233/CBM-150562 26756615 PMC13016453

[10] Kimura K Matsumoto S Harada T Morii E Nagatomo I Shintani Y. ARL4C is associated with initiation and progression of lung adenocarcinoma and represents a therapeutic target. Cancer Sci. (2020) 111:951–61. 10.1111/cas.14303 31925985 PMC7060486

[11] Yuan JS Reed A Chen F Stewart CN Jr Statistical analysis of real-time PCR data. BMC Bioinform. (2006) 7:85 10.1186/1471-2105-7-85 PMC1395339 16504059

[12] Lin XD Chen SQ Qi YL Zhu JW Tang Y Lin JY. Overexpression of thrombospondin-1 in stromal myofibroblasts is associated with tumor growth and nodal metastasis in gastric carcinoma. J Surg Oncol. (2012) 106:94–100. 10.1002/jso.23037 22231149

[13] Mok TS Wu YL Thongprasert S Yang CH Chu DT Saijo N Gefitinib or carboplatin-paclitaxel in pulmonary adenocarcinoma. N Engl J Med. (2009) 361:947–57. 10.1056/NEJMoa0810699 19692680

[14] Lu X Yu L Zhang Z Ren X Smaill JB Ding K. Targeting EGFR(L858R/T790M) and EGFR(L858R/T790M/C797S) resistance mutations in NSCLC: current developments in medicinal chemistry. Med Res Rev. (2018) 38:1550–81. 10.1002/med.21488 29377179

[15] Wang Q Yang S Wang K Sun SY. MET inhibitors for targeted therapy of EGFR TKI-resistant lung cancer. J Hematol Oncol. (2019) 12:63. 10.1186/s13045-019-0759-9 31227004 PMC6588884

[16] de Langen AJ Jebbink M Hashemi SMS Kuiper JL de Bruin-Visser J Monkhorst K. Trastuzumab and paclitaxel in patients with EGFR mutated NSCLC that express HER2 after progression on EGFR TKI treatment. Br J Cancer. (2018) 119:558–64. 10.1038/s41416-018-0194-7 30061586 PMC6162232

[17] Fujii S Matsumoto S Nojima S Morii E Kikuchi A. Arl4c expression in colorectal and lung cancers promotes tumorigenesis and may represent a novel therapeutic target. Oncogene. (2015) 34:4834–44. 10.1038/onc.2014.402 25486429

[18] Harada T Matsumoto S Hirota S Kimura H Fujii S Kasahara Y. Chemically modified antisense oligonucleotide against ARL4C inhibits primary and metastatic liver tumor growth. Mol Cancer Ther. (2019) 18:602–12. 10.1158/1535-7163.MCT-18-0824 30647122

[19] Stewart DJ Wnt signaling pathway in non-small cell lung cancer. J Natl Cancer Inst. (2014) 106:djt356 10.1093/jnci/djt356 24309006

[20] Cao S Wang Z Gao X He W Cai Y Chen H. FOXC1 induces cancer stem cell-like properties through upregulation of beta-catenin in NSCLC. J Exp Clin Cancer Res. (2018) 37:220. 10.1186/s13046-018-0894-0 30189871 PMC6127900

[21] Suzuki M Shigematsu H Nakajima T Kubo R Motohashi S Sekine Y. Synchronous alterations of Wnt and epidermal growth factor receptor signaling pathways through aberrant methylation and mutation in non small cell lung cancer. Clin Cancer Res. (2007) 13:6087–92. 10.1158/1078-0432.CCR-07-0591 17947472

[22] Li K Mo C Gong D Chen Y Huang Z Li Y. DDX17 nucleocytoplasmic shuttling promotes acquired gefitinib resistance in non-small cell lung cancer cells via activation of beta-catenin. Cancer Lett. (2017) 400:194–202. 10.1016/j.canlet.2017.02.029 28259822

[23] Wang J Zhou P Wang X Yu Y Zhu G Zheng L. Rab25 promotes erlotinib resistance by activating the beta1 integrin/AKT/beta-catenin pathway in NSCLC. Cell Prolif. (2019) 52:e12592. 10.1111/cpr.12592 30848009 PMC6536583

[24] Groner B von Manstein V. Jak Stat signaling and cancer: opportunities, benefits and side effects of targeted inhibition. Mol Cell Endocrinol. (2017) 451:1–14. 10.1016/j.mce.2017.05.033 28576744

[25] Chai EZ Shanmugam MK Arfuso F Dharmarajan A Wang C Kumar AP. Targeting transcription factor STAT3 for cancer prevention and therapy. Pharmacol Ther. (2016) 162:86–97. 10.1016/j.pharmthera.2015.10.004 26478441

[26] Phesse TJ Buchert M Stuart E Flanagan DJ Faux M Afshar-Sterle S. Partial inhibition of gp130-Jak-Stat3 signaling prevents Wnt-beta-catenin-mediated intestinal tumor growth and regeneration. Sci Signal. (2014) 7:ra92. 10.1126/scisignal.2005411 25270258

[27] Wu J Keng VW Patmore DM Kendall JJ Patel AV Jousma E. Insertional Mutagenesis Identifies a STAT3/Arid1b/beta-catenin Pathway Driving Neurofibroma Initiation. Cell Rep. (2016) 14:1979–90. 10.1016/j.celrep.2016.01.074 26904939 PMC4782770

[28] Song X Wang S Li L. New insights into the regulation of Axin function in canonical Wnt signaling pathway. Protein Cell. (2014) 5:186–93. 10.1007/s13238-014-0019-2 24474204 PMC3967064

[29] Bhattacharya R Mitra T Ray Chaudhuri S Roy SS. Mesenchymal splice isoform of CD44 (CD44s) promotes EMT/invasion and imparts stem-like properties to ovarian cancer cells. J Cell Biochem. (2018) 119:3373–83. 10.1002/jcb.26504 29130517

[30] Gennaro VJ Stanek TJ Peck AR Sun Y Wang F Qie S. Control of CCND1 ubiquitylation by the catalytic SAGA subunit USP22 is essential for cell cycle progression through G1 in cancer cells. Proc Natl Acad Sci USA. (2018) 115:E9298–307. 10.1073/pnas.1807704115 30224477 PMC6176615

[31] Choi YJ Kim N Lee HS Park SM Park JH Yoon H. Expression of leucine-rich repeat-containing G-protein coupled receptor 5 and CD44: potential implications for gastric cancer stem cell marker. J Cancer Prevent. (2016) 21:279–87. 10.15430/JCP.2016.21.4.279 28053963 PMC5207613

[32] Han B Zhou B Qu Y Gao B Xu Y Chung S. FOXC1-induced non-canonical WNT5A-MMP7 signaling regulates invasiveness in triple-negative breast cancer. Oncogene. (2018) 37:1399–408. 10.1038/s41388-017-0021-2 29249801 PMC5844802

