# Supplementary material for: The Role of ARL4C in Erlotinib Resistance: Activation of the Jak2/Stat 5/β-Catenin Signaling Pathway
Source: Front Oncol. 2020 Oct 28;10:585292. doi: 10.3389/fonc.2020.585292 (PMC7657464; doi:10.3389/fonc.2020.585292)
Supplement: Supplementary file 1 [file Table_1.DOC]

| **Supplementary Table 1. The gene list and primers for RT-qPCR** | | |
| --- | --- | --- |
| **Primer** | **Sequence** | **Length(bases)** |
| **ARL4C** |  |  |
| F-primer | 5’-CTACCGGCTCAAGTTCAACG-3’ | 20 |
| R-primer | 5’-CGAGTCCACCACGTAGATGA-3’ | 20 |
| **CTNNB1** |  |  |
| F-primer | 5’-TCCCACTAATGTCCAGCGTT-3’ | 20 |
| R-primer | 5’-ATGGACCATAACTGCAGCCT-3’ | 20 |
| **Axin2** |  |  |
| F-primer | 5’-TCAAGACGGTGCTTACCTGT-3’ | 20 |
| R-primer | 5’-TGCTGCTTCTTGATGCCATC-3’ | 20 |
| **CD44** |  |  |
| F-primer | 5’-TCCTCACATCCAACACCTCC-3’ | 20 |
| R-primer | 5’-GCTGCTCACGTCATCATCAG-3’ | 20 |
| **Ccnd1** |  |  |
| F-primer | 5’-GCATGTTCGTGGCCTCTAAG-3’ | 20 |
| R-primer | 5’-CGTGTTTGCGGATGATCTGT-3’ | 20 |
| **Lgr5** |  |  |
| F-primer | 5’-TATCGTCCAACCTCCTGTCG-3’ | 20 |
| R-primer | 5’-AGGCATTCTCACACACTCCA-3’ | 20 |
| **MMP7** |  |  |
| F-primer | 5’-TGGGGAACTGCTGACATCAT-3’ | 20 |
| R-primer | 5’-CCCTAGACTGCTACCATCCG-3’ | 20 |
| **GAPDH** |  |  |
| F-primer | 5’-CCAGAACATCATCCCTGCCT-3’ | 20 |
| R-primer | 5‘-CCTGCTTCACCACCTTCTTG-3’ | 20 |
