# Supplementary material for: The Role of ARL4C in Erlotinib Resistance: Activation of the Jak2/Stat 5/β-Catenin Signaling Pathway
Source: Front Oncol. 2020 Oct 28;10:585292. doi: 10.3389/fonc.2020.585292 (PMC7657464; doi:10.3389/fonc.2020.585292)
Supplement: Supplementary file 2 [file Table_2.DOC]

| **Supplementary Table 2. Patients clinical-pathological characteristics and EGFR status** | | | | | | |
| --- | --- | --- | --- | --- | --- | --- |
| **PATIENT_ID** | **AGE** | **SEX** | **HISTOLOGY** | **TNM** | **SMOKING_HISTORY** | **EGFR Detection** |
| Case1 | 65 | Male | Invasive adenocarcinoma | T2aN0M0 IB | Ex-Smoker | EGRF wild type |
| Case2 | 69 | Male | Invasive adenocarcinoma | p3TN2M0 IIIa | Ex-Smoker | EGRF wild type |
| Case3 | 41 | Femal | Invasive adenocarcinoma | pT1aN0M0 Ia | Recent Ex-Smoker | EGRF wild type |
| Case4 | 69 | Male | Squamous cell carcinoma | T2N2M0 IIIa | Recent Ex-Smoker | EGRF wild type |
| Case5 | 63 | Male | Invasive adenocarcinoma | T2aN0M0 Ib | Ex-Smoker | EGFR L861Q |
| Case6 | 49 | Male | Invasive adenocarcinoma | T2aN0M0Ⅰb | Recent Ex-Smoker | EGRF wild type |
| Case7 | 56 | Femal | Adenosquamous carcinoma | T4N2M0 IIIb | Ex-Smoker | EGFR S768I |
| Case8 | 55 | Femal | Invasive adenocarcinoma | T1bN0M0Ⅰa | Ex-Smoker | EGFR 19DEL |
| Case9 | 51 | Femal | Invasive adenocarcinoma | T3N0MO IIB | Ex-Smoker | EGRF wild type |
| Case10 | 57 | Male | Invasive adenocarcinoma | T2aN2M0 IIIA | Recent Ex-Smoker | EGRF wild type |
| Case11 | 58 | Male | Carcinosarcoma | T1bN2M0 IIIA | Recent Ex-Smoker | EGRF wild type |
| Case12 | 52 | Femal | Invasive adenocarcinoma | T2aN0M0 Ib | Never Smoked | EGFR 19DEL |
| Case13 | 63 | Femal | Invasive adenocarcinoma | T2aN0M0 IB | Ex-Smoker | EGRF wild type |
| Case14 | 64 | Male | Invasive adenocarcinoma | T2bN0M0 IB | Ex-Smoker | EGRF wild type |
| Case15 | 70 | Femal | Invasive adenocarcinoma | T2aN2M0 IIIa | Recent Ex-Smoker | EGRF wild type |
| Case16 | 64 | Femal | Invasive adenocarcinoma | T2aN1M0 IIA | Ex-Smoker | EGFR L858R |
| Case17 | 61 | Femal | Invasive adenocarcinoma | T2aN1M0 IIA | Ex-Smoker | EGFR 19DEL |
| Case18 | 46 | Male | Invasive adenocarcinoma | T1aN2M0 IIIa | Ex-Smoker | EGRF wild type |
| Case19 | 73 | Male | Invasive adenocarcinoma | T2N0M0 IB | Ex-Smoker | EGFR L858R |
| Case20 | 72 | Femal | Invasive adenocarcinoma | T1aN0M0 IA | Ex-Smoker | EGFR 19DELl and T790M |
| Case21 | 58 | Femal | Invasive adenocarcinoma | T2bN2M0 IIIA | Never Smoked | EGFR 19DEL |
| Case22 | 65 | Male | Squamous cell carcinoma | T2N0M0 IB | Never Smoked | EGRF wild type |
| Case23 | 67 | Femal | Invasive adenocarcinoma | T2bN2M0 IIIA | Ex-Smoker | EGFR L858R |
| Case24 | 83 | Male | Squamous cell carcinoma | T1N0M0 IA | Never Smoked | EGRF wild type |
| Case25 | 58 | Femal | Invasive adenocarcinoma | T2N2M0 IIIA | Never Smoked | EGRF wild type |
| Case26 | 59 | Male | Invasive adenocarcinoma | T1bN0M0 Ia | Never Smoked | EGRF wild type |
| Case27 | 60 | Femal | Invasive adenocarcinoma | T4N0M0 IIIA | Ex-Smoker | EGFR G719A |
| Case28 | 61 | Male | Squamous cell carcinoma | T2aN0M0 Ib | Recent Ex-Smoker | EGRF wild type |
| Case29 | 60 | Femal | Invasive adenocarcinoma | T2aN2M0 IIIA | Never Smoked | EGRF wild type |
| Case30 | 71 | Femal | Invasive adenocarcinoma | T1cN1M0 IIB | Never Smoked | EGRF wild type |
| Case31 | 52 | Male | Invasive adenocarcinoma | T2aN1M0 Iia | Never Smoked | EGFR 19DEL |
| Case32 | 54 | Femal | Invasive adenocarcinoma | T2N2M0 IIIa | Never Smoked | EGFR L861Q |
| Case33 | 68 | Male | Invasive adenocarcinoma | T2aN0M0，IB | Ex-Smoker | EGFR L858R |
| Case34 | 60 | Male | Invasive adenocarcinoma | T1aN0M0 Ia | Recent Ex-Smoker | EGRF wild type |
| Case35 | 70 | Male | Invasive adenocarcinoma | T1bN0M0 IA2 | Ex-Smoker | EGFR 19DEL |
| Case36 | 72 | Femal | Invasive adenocarcinoma | T2bN2M0 IIIA | Never Smoked | EGFR 19DEL |
| Case37 | 53 | Male | Squamous cell carcinoma | T1aN0M0 IA | Never Smoked | EGRF wild type |
| Case38 | 37 | Femal | Invasive adenocarcinoma | T1CN2M1b IV A | Never Smoked | EGFR 19DEL |
| Case39 | 55 | Male | Invasive adenocarcinoma | T 1bN0M0 IA | Recent Ex-Smoker | EGFR 19DEL |
| Case40 | 48 | Male | Invasive adenocarcinoma | T2N2 M0 IIIA | Ex-Smoker | EGFR 20-INS |
| Case41 | 62 | Male | Invasive adenocarcinoma | T1cN0M0 IA | Ex-Smoker | EGRF wild type |
| Case42 | 67 | Femal | Invasive adenocarcinoma | T2aN0M0 IB | Never Smoked | EGFR 19DEL |
